# The Potential of *Aloe vera* as a Caries Prevention Agent in the Future: A Scoping Review

**DOI:** 10.3390/jcm15124744

**Published:** 2026-06-18

**Authors:** Irmaleny Irmaleny, Denny Nurdin, Indra Primathena, Huwaina Abd Ghani

**Affiliations:** 1 Department of Conservative Dentistry, Faculty of Dentistry, Universitas Padjadjaran, Bandung 40132, Indonesia; 2 Restorative Dentistry and Endodontic, School of Dental Sciences, Health Campus, Universiti Sains Malaysia, Kota Bharu 16150, Malaysia

**Keywords:** *Aloe vera*, caries prevention, dental caries, dentistry

## Abstract

Untreated dental caries in permanent teeth is the most frequent disease of all 371 diseases and traumas assessed by the Global Burden of Disease Study in 2021, and there are reported to be 2.24 billion cases worldwide. Demineralization is a disintegration process of minerals and apatite crystals in hard tissue, provoked by biofilm activities, dietary factors, and the micro-oral environment—the three main mechanisms of dental caries. Restoration of mineral ions in the crystal structure is defined as remineralization. Remineralization enables the deposition of new minerals within the crystal structure of demineralized enamel, aiming to increase mineral production. Environments suitable for remineralization and inhibiting demineralization could be created by using a caries prevention agent. **Objectives:** Providing scientific evidence regarding *Aloe vera* as an alternative agent for caries prevention. **Materials and Method:** The method used in this study is a scoping review, utilizing the PRISMA-ScR as a guideline to conduct article screening and further analysis, following a thematic analysis approach. Database searches were conducted in PubMed, EBSCOhost, and ScienceDirect, based on the keywords generated. **Results:** A total of 13 articles were gathered for further analysis. **Conclusions:** *Aloe vera* shows promising preliminary potential, but further standardized in vivo and randomized clinical studies are necessary to confirm its remineralizing efficacy and clarify its mechanisms of action as a cavity prevention agent. **Clinical Relevance:** Using *Aloe vera* as an alternative caries prevention agent.

## 1. Introduction

Dental caries is a notable oral health problem in communities worldwide. The risk of dental caries increases with the continuous consumption of carbohydrates and sugar, resulting in increased acidity and disruption of the oral microbial balance. Excessive acid production leads to a dysbiotic shift in the bacteria, compromising the biofilm, leading to tooth demineralization and dental caries [1]. Untreated dental caries in permanent teeth is the most frequent health problem among the 371 diseases and traumas evaluated by the Global Burden of Disease Study in 2021, with a total of 2.24 billion cases worldwide [2]. According to the Indonesian Health Survey conducted by the Minister of Health of the Republic of Indonesia, the prevalence of dental caries was reported to be relatively high, at 82.8%.

Demineralization is a complex process caused by biofilm activities, dietary factors, and the micro-oral environment, which are the primary mechanisms of dental caries [3]. Demineralization is indicated by the release of mineral ions from hydroxyapatite crystals of the body’s hard tissues, i.e., enamel, dentin, cementum, and bone. The restoration of mineral ions in hydroxyapatite crystals is referred to as remineralization [4]. Remineralization originating from external sources enables the deposition of new minerals into the crystal structure of demineralized enamel, aiming to increase the mineral content [5,6]. Environments suitable for remineralization and inhibiting demineralization could be created by using a caries prevention agent [7].

One of the most widely used and studied tooth remineralization treatments is Casein Phosphopeptide–Amorphous Calcium Phosphate (CPP-ACP). However, CPP-ACP has a drawback—it poses a high risk for lactose-intolerant individuals and is expensive [8,9]. Nature-based remineralization agents have emerged as an alternative to common chemical-based agents, one of which is *Aloe vera* [10]. There are around 250 species of *Aloe vera* available globally. *Aloe vera* vegetates well in hot and humid areas, such as the Pacific Rim countries (Malaysia, China, Indonesia, etc.) [11]. *Aloe vera* is the most scientifically approved and considered agent among the various herbal agents available nowadays [11]. *Aloe vera* is commonly used as an agent to stimulate wound healing, moisturize the skin, and provide anti-aging and laxative properties, amongst others. [11]. In the field of dentistry, *Aloe vera* has not been widely studied, particularly regarding its remineralization properties for teeth. Based on the current state of affairs, this study was conducted to discuss and provide scientific evidence supporting the use of *Aloe vera* as an alternative agent for inducing cavity prevention in order to provide a suitable environment for promoting remineralization and inhibiting demineralization.

## 2. Method

A scoping review is a type of evidence synthesis that aims to systematically identify and map the breadth of available evidence on a particular topic, field, concept, or issue, regardless of source, for a specific context. A scoping review approach can sharpen the core concepts or definitions in the literature, as well as identify the characteristics and key factors related to an idea, including those relevant to methodological research [12]. In conducting a scoping review, the study is performed by identifying journals in electronic databases according to inclusion and exclusion criteria compiled based on the Population, Concept, and Context (PCC) concept. Data extraction in this study followed the Preferred Reporting Items for Systematic Reviews and Meta-Analyses Extension for Scoping Reviews (PRISMA-ScR). The data analysis in this study was thematic analysis, i.e., an analysis that evaluates text excerpts and correlates them to research questions [13,14].

**Figure 1:**
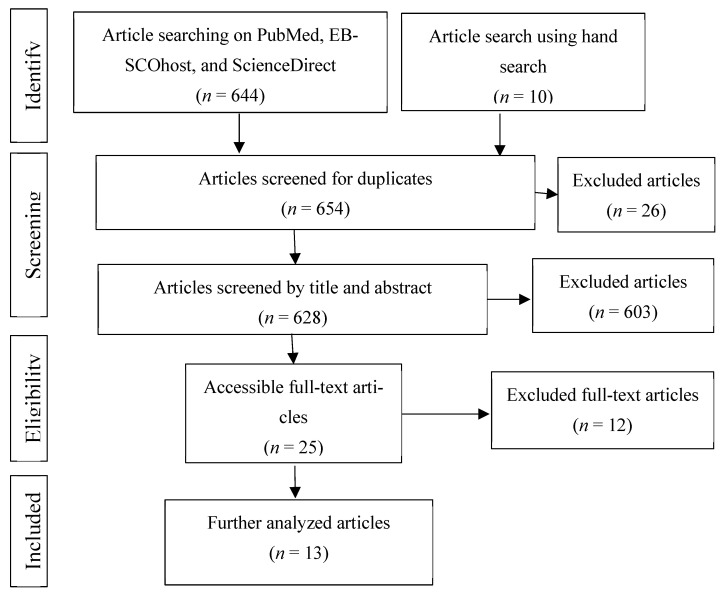
PRISMA-ScR flow chart.

The identification and searching of electronic databases were conducted using the following keywords: (“*Aloe vera*” AND “dentistry”, “*Aloe vera*” AND “remineralization, *Aloe vera*” AND “bioactive agent”), and (“*Aloe vera*” AND “dental caries”). The search was conducted on EBSCOhost, PubMed, and ScienceDirect (Table 1) according to the PRISMA-ScR guidelines and a publication year filter (2015–2025). The steps consisted of article screening, eligibility examination, and finalization of the included articles, as presented in Figure 1. The article selection for further analysis was conducted based on the inclusion and exclusion criteria (Table 2). The article selection stage consisted of duplicate checking, title and abstract screening, and full-text reading. A PRISMA-ScR checklist is provided in Supplementary Table S1.

Article screening on the electronic databases generated 644 articles (92 from EBSCOhost, 201 from PubMed, and 351 from ScienceDirect). Ten articles were found via the hand search method. All 654 articles were screened for duplicates, and 26 were excluded. Following the duplicate screening, abstract and title screening was conducted, and 603 articles were excluded. The 25 remaining articles were read in full text, resulting in the exclusion of 12 articles. After undergoing the stages of the PRISMA-ScR approach, a total of 13 articles were selected for further analysis.

## 3. Result

A total of 13 articles regarding *Aloe vera*’s potential as a caries prevention agent were analyzed further. The articles were studied comprehensively. Data extraction of author, country of origin, publication year, study design, medication form of *Aloe vera*, results, and conclusions is presented in Table 3.

## 4. Discussion

*Aloe vera* is a short-stemmed and cactus-like plant with dagger-shaped, spiny, and serrated green leaves containing a clear gel that possesses remarkable antibacterial, antifungal, and antiviral properties [17]. *Aloe vera* is commonly used in dentistry due to its effects on oral mucosal diseases, pulpotomy, and alveolar osteitis; its ability to inhibit Enterococcus faecalis; its potential as a root canal filling material in primary teeth; its applications in periodontology, gutta-percha disinfection, and denture adhesive formulation; and as a disinfectant for irrigation [20,28]. With the rise in demand for natural products, research on remineralization and caries prevention has increased, particularly regarding *Aloe vera*. *Aloe vera* can be a suitable alternative due to its beneficial effects, wide availability, low cost, and lack of known side effects [26].

*Aloe vera* has been evaluated as a caries prevention agent from several perspectives, including its antibacterial properties, increase in microhardness, remineralization ability, and decrease in plaque score. According to Bhati et al., *Aloe vera* contains specific components, including anthraquinones, dihydroxyanthraquinones, and saponins, which are considered antimicrobial components that inhibit protein synthesis in bacterial cells [18]. *Aloe vera*’s antibacterial properties, according to Hajiahmadi et al., can inhibit the growth of *Streptococcus mutans* and *Lactobacillus*, which are the bacteria that cause dental caries, and *Aloe vera* was also reported to yield a better antimicrobial effect compared to xylitol and CPP-ACP [24]. Consistent with the previous study, Prabhakar et al. and Patri et al. stated that *Aloe vera* could be an alternative for disinfecting cavities after Atraumatic Restorative Treatment (ART). The two studies verified the effectiveness of this natural antibacterial agent in preventing secondary caries and increasing the longevity of restoration [17,25].

Regarding remineralization ability, Haddad et al. stated that *Aloe vera* gel can exhibit a similar remineralization effect to toothpaste with 1450 ppm fluoride. The polyphenols present in *Aloe vera* gel are active components that contribute to remineralization in teeth [15]. Conforming to Haddad et al., the remineralization effect of *Aloe vera* was also evidenced in a study by Yikici et al., which reported that remineralization in early enamel lesions due to *Aloe vera* yielded a similar effect to that of 1450 ppm fluoridated toothpaste [20]. *Aloe vera*’s remineralization effect was also reported by a study by Gandhi et al. and Silva et al., stating that *Aloe vera* could induce remineralization in white spot lesions [19,22]. Silva et al. revealed that the use of *Aloe vera*-based toothpaste was not more abrasive and was able to reduce the risk of fluorosis [22]. According to Gandhi et al., *Aloe vera* could be combined in toothpaste or mouthwash to develop safe, effective, and economical products [19]. Al-Shaibani et al. reported an increase in enamel microhardness following *Aloe vera* application in their study; therefore, it can be affirmed that *Aloe vera* is a safe and effective agent for preventing dental caries [23]. According to Srisomboon et al., the addition of propolis extract to *Aloe vera* could increase the dentin remineralization effect in experiments using artificial saliva [27].

In addition to its remineralization and antibacterial properties, *Aloe vera* has also been shown to reduce plaque and support gingival health, as reported by Hajmonari et al., Yeturu et al., and Vajrabhaya et al. [16,21,26]. According to Hamonari et al., *Aloe vera* was proven to be as effective as chlorhexidine in reducing plaque and gingivitis. This finding suggests that *Aloe vera* may be an alternative in preventing gingivitis and offers a new choice for individuals seeking to maintain oral health with natural products [16]. According to Vajrabhaya et al., *Aloe vera* in toothpaste may reduce gingival inflammation more effectively than standard toothpaste [21]. In accordance with this finding, Yeturu et al. stated that *Aloe vera* may be a suitable and economical option to replace chlorhexidine as a mouthwash for decreasing plaque and preventing gingivitis [26].

Further study is suggested to be conducted regarding the increase in remineralization and inhibit demineralization effects of *Aloe vera*, aiming to validate the sustainability of its active component and its mechanism as caries prevention agent. This study aimed to provide scientific evidence supporting the use of *Aloe vera* as a caries prevention agent. However, this study was limited by the various articles, differing design studies, and distinct forms of *Aloe vera* medication used in the studies.

## 5. Conclusions

*Aloe vera* shows promising preliminary potential, but further standardized in vivo and randomized clinical studies are necessary to confirm its remineralizing efficacy and clarify its mechanisms of action as a cavity prevention agent.

## Figures and Tables

**Table 1:** Article search in the databases using the keywords.

Search Engine	Keywords	Number of Articles
EBSCOhost	“*Aloe vera*” AND “dentistry, *Aloe vera*” AND “remineralization, *Aloe vera*” AND “bioactive agent”, “*Aloe vera*” AND “dental caries”	92 articles
PubMed	“*Aloe vera*” AND “dentistry, *Aloe vera*” AND “remineralization, *Aloe vera*” AND “bioactive agent”, “*Aloe vera*” AND “dental caries”	201 articles
ScienceDirect	“*Aloe vera*” AND “dentistry, *Aloe vera*” AND “remineralization, *Aloe vera*” AND “bioactive agent”, “*Aloe vera*” AND “dental caries”	351 articles

**Table 2:** Inclusion and exclusion criteria.

Inclusion Criteria	Exclusion Criteria
1. Articles published from 2015 to 2025 consisting of trials and experiments	1. Review articles (all types of review, including meta-analyses)
2. Articles in Indonesian and English available in the databases	2. Identical articles in different databases (duplicates)
3. Articles reporting on *Aloe vera*’s effect as a caries prevention agent	
4. Open-access full-text articles	

**Table 3:** *Aloe vera*’s effect as a caries prevention agent.

Author, Country (Publication Year)	Title	Study Design	Medication Form of *Aloe vera*	Results of the Study	Conclusion
Haddad et al., Lebanon (2021) [15]	Comparison of the Remineralizing Effect of Brushing with *Aloe vera* versus Fluoride Toothpaste	In vitro	*Aloe vera* non-fluoridated/fluoridated toothpaste, and *Aloe vera* gel	40 premolars and molars were cleaned and inspected, after which demineralization was performed. The teeth were divided into four groups: Group A (1450 ppm fluoride toothpaste), Group B (AV 1000 ppm fluoride toothpaste), Group C (AV non-fluoridated toothpaste), and Group D (AV leaf gel). The results showed that the non-fluoridated group yielded the lowest result, while the other groups yielded similar results.	Remineralization induced by 1000 ppm fluoridated AV toothpaste reported similar results to 1450 ppm *fluoride toothpaste*
Hamonari et al., Iraq (2024) [16]	Effectiveness of Chlorhexidine and *Aloe vera* Mouthwash in Patients with Periodontal Disease: A Randomized Controlled Trial	A Randomized Controlled Trial	*Aloe vera* mouthwash	270 volunteers with moderate or severe gingivitis (144 females and 126 males, aged 18–45) were divided into three groups: Group A (*Aloe vera* mouthwash), Group B (water), and Group C (0.2% chlorhexidine). The result showed a decrease in plaque score and gingivitis index in the *Aloe vera* and chlorhexidine groups.	*Aloe vera* was proven to be as effective as chlorhexidine in reducing plaque and gingivitis
Prabhakar et al., India (2015) [17]	Cavity disinfection in minimally invasive dentistry—comparative evaluation of *Aloe vera* and propolis: A randomized clinical trial	A Randomized Clinical Trial, In vivo	*Aloe vera* extract (ethanol extract from *Aloe vera* leaves)	Ten children aged 5–12 with a minimum of three teeth with occlusal or occlusoproximal lesions indicated for Atraumatic Restorative Treatment (ART) had their dentin taken as samples. The dentin samples underwent microbial tests. The samples were divided into three groups: Group 1 (water), Group 2 (propolis extract), and Group 3 (*Aloe vera* extract). The results showed a decrease in the number of bacteria after application post-ART.	*Aloe vera* and propolis in the form of extract had the potential for cavity disinfection
Bhati et al., India (2015) [18]	Evaluation of antimicrobial efficacy of *Aloe vera* and Meswak containing dentifrices with fluoridated dentifrice: An in vivo study	In Vivo	*Aloe vera* dentifrice	60 children aged 6–12 with the DMF/def 0 index score were divided into four groups: Group A (without intervention), Group B (fluoridated toothpaste), Group C (toothpaste with *Aloe vera*), and Group D (toothpaste with meswak). The results showed a decrease in the number of bacteria in the *Aloe vera*, meswak, and fluoridated toothpaste groups.	Herbal toothpaste containing *Aloe vera* and meswak can be a safe alternative for fluoridated toothpaste in terms of the microbial effect
Gandhi et al., India (2022) [19]	Comparison of the Remineralisation Potential between Flaxseed Paste, *Aloe vera* Gel, and Fluoride Toothpaste on Artificially Created White Spot Lesions around Orthodontic Brackets: An In-vitro Study	In Vitro	*Aloe vera* gel	48 premolars were demineralized and divided into four groups: Group 1 (without intervention), Group 2 (flaxseed paste), Group 3 (*Aloe vera* gel), and Group 4 (fluoridated toothpaste). The results showed that the *Aloe vera* gel yielded the highest result in surface hardness compared to the other groups	All groups showed an increase in minerals. However, *Aloe vera* showed promising results in remineralizing white spot lesions
Yikici et al., Turkey (2022) [20]	Remineralization Activities of Toothpastes with and without *Aloe vera* with Different Ratios of Fluoride on Demineralized Enamel: An In-vitro Study	In Vitro	*Aloe vera* gel (gel extracted from *Aloe vera* leaves), *Aloe vera* combined with toothpaste (fluoridated and non-fluoridated)	72 third molars were inspected and divided into two major groups, then four minor groups: Group A1 (without intervention), Group A2 (non-fluoridated toothpaste), Group A3 (toothpaste with 1100 ppm fluoride), Group A4 (toothpaste with 1450 ppm fluoride), Group B1 (*Aloe vera* gel), Group B2 (non-fluoridated toothpaste combined with *Aloe vera*), Group B3 (toothpaste with 1000 ppm fluoride combined with *Aloe vera*), and Group B4 (toothpaste with 1440 ppm fluoride combined with *Aloe vera*). The results showed that, statistically, the fluoridated toothpaste combined with *Aloe vera* yielded a higher microhardness.	Toothpaste with 1450 ppm fluoride combined with *Aloe vera* provided a good remineralization effect, and *Aloe vera* could possess a synergistic effect with sodium monofluorophosphate formulations
Vajrabhaya et al., Thailand (2024) [21]	Efficacy of a Herbal Toothpaste During Active Periodontal Treatment: A Clinical Study	Clinical Study	Toothpaste containing *Aloe vera*	54 patients with periodontitis were divided into three groups: Group 1 (toothpaste with *Aloe vera*), Group 2 (toothpaste with sodium bicarbonate), and Group 3 (standard toothpaste). The results showed a more significant decrease in clinical attachment loss in the *Aloe vera* group compared to the other two groups.	Toothpaste with *Aloe vera* can reduce gingival inflammation, probing depth, and clinical attachment loss compared to standard toothpaste in periodontitis patients
Silva et al., Brazil (2016) [22]	Effects of fluoride and *Aloe vera* tooth gel in artificial white spot lesions in vitro	In Vitro	*Aloe vera tooth gel*	20 bovine teeth were inspected and prepared. The teeth were divided into two groups: Group 1 (fluoridated toothpaste) and Group 2 (*Aloe vera* gel). The results showed no significant difference between the two groups in terms of microhardness.	Both toothpastes with fluoride or *Aloe vera* can effectively increase the superficial microhardness of artificial white spot lesions
Al-Shaibani et al., Iraq (2023) [23]	The Impact of *Aloe vera* Gel on Remineralization of The Tooth and Its Effect against Enterococcus Faecalis: An In Vitro Study	In Vitro	*Aloe vera* gel	Ten permanent molars were demineralized and divided into two groups: Group 1 (water) and Group 2 (*Aloe vera* gel). The results showed significant differences between the *Aloe vera* and water-only groups in terms of density and microhardness.	*Aloe vera* gel can be used for caries prevention due to its effectiveness and safety
Hajiahmadi et al., Iran (2021) [24]	Comparative Evaluation of Antibacterial Effect of Propolis and *Aloe vera*, Xylitol, and CPP-ACP Gels on Streptococcus mutans and Lactobacillus in Vitro	In Vitro	*Aloe vera* and propolis gel	Bacterial cultures were prepared to represent all the materials to be tested. The groups were divided into propolis and *Aloe vera* gel, CPP-ACP, xylitol, and 1000 ppm fluoride. Each culture had one positive control and one negative control. The results showed the best bacterial inhibition was discovered in the gel containing propolis and *Aloe vera* against Streptococcus mutans and Lactobacillus.	Propolis and *Aloe vera* gel exhibited superior antimicrobial effects compared to other gels, and these effects were evident even at low concentrations
Patri et al., India (2017) [25]	Role of Herbal Agents—Tea Tree Oil and *Aloe vera* as Cavity Disinfectant Adjuncts in Minimally Invasive Dentistry- An In vivo Comparative Study	In Vivo	*Aloe vera* gel	Ten patients with a minimum of one tooth with occlusal or occlusoproximal lesions indicated for ART were divided into four groups: Group 1 (2% chlorhexidine), Group 2 (tea tree oil), Group 3 (*Aloe vera* gel), and Group 4 (water). The results showed that the chlorhexidine group yielded higher antibacterial activity than the other two ingredients.	Natural antibacterials, e.g., tea tree oil and *Aloe vera* gel, can be effective options for cavity disinfection, which can minimize secondary caries and enable long-lasting fillings
Yeturu et al., India (2015) [26]	Effect of *Aloe vera*, chlorine dioxide, and chlorhexidine mouth rinses on plaque and gingivitis: A randomized controlled trial	A Randomized Controlled Trial	*Aloe vera* mouthwash	90 patients undergoing fixed orthodontic treatment were divided into three groups: Group 1 (*Aloe vera*), Group 2 (chlorhexidine), and Group 3 (chlorine dioxide). Results showed a significant reduction in plaque in all three groups	Chlorine dioxide and *Aloe vera* can be economical alternatives to chlorhexidine
Srisomboon et al., Thailand (2024) [27]	The in vitro assessment of rheological properties and dentin remineralization of saliva substitutes containing propolis and *Aloe vera* extracts	In Vitro	Artificial saliva with propolis and *Aloe vera*	Six groups of saliva substitutes, each containing different concentrations of propolis and *Aloe vera*, and a control group containing only water. The results showed that adding propolis to *Aloe vera* extract increased the mineral content.	The addition of propolis extract increased dentin remineralization

## Data Availability

Research data available by contacting irmaleny@unpad.ac.id.

## Supplementary Materials

The following supporting information can be downloaded at: https://www.mdpi.com/article/10.3390/jcm15124744/s1, Table S1: Preferred Reporting Items for Systematic reviews and Meta-Analyses extension for Scoping Reviews (PRISMA-ScR) Checklist [14].
